# Tungsten and Its Dental Uses

**Published:** 1913-01-15

**Authors:** L. E. Custer

**Affiliations:** Dayton, Ohio


### TUNGSTEN AND ITS DENTAL USES.

BY L. E. CUSTER, A.M., D.D.S., DAYTON, OHIO.

I wish to call attention to a comparatively new metal that has some valuable properties when used in dentistry. This metal is known as Tungsten. It does not occur as such in nature, but in the form of its compounds. This element is widely distributed, and is most frequently found in the form of Wolframite, which is a tungstate of iron and manganese. It is also found in the form of Sheelite or calcium tungstate. The most of this comes from Boulder County, Colorado.

The properties which make this remarkable metal are its high melting point, its resistance to ordinary reagents, and its hardness. Its melting point is 5400 degrees Fahrenheit, almost twice that of platinum. It comes just within the range of the electric arc, the highest heat that can be produced. It is because of the above fact, that it has been so difficult to produce it in wrought metallic form. The first tungsten lamp filaments were made of a powdered form of tungsten which were easily broken when subjected to vibration. Those which are now made are from the drawn wire. In this connection, it might be well to explain the advantages of tungsten as a lamp filament. The light of an incandescent lamp is due to the safe heat to which the filament can be raised. You are all familiar with the carbon filament, and you have noticed that it can burn with safety at what we call a white heat. If the voltage is raised, it reaches a dazzling super-white heat which it can stand for but a moment before burning out. At this point, the amount of light emitted is about squared as to the rise of current. Here is the advantage of tungsten over carbon: its high melting point enables it to be brought to a much higher heat than carbon, thus emitting more light with but little more current. The tungsten lamp is about twice as efficient, and therefore more economical than a carbon lamp.

The high melting point of tungsten suggests its use for electric ovens, but while it resists all single chemical agents, it oxidizes when brought to a high heat. An electric oven can be made of tungsten wire if the wire is wound upon a refractory crucible, and this crucible encased in a larger one through which a stream of hydrogen gas is flowing. The hydrogen will not explode when used in this manner, but simply burns as it escapes at the upper opening. Another method suggests itself at the present time as follows: the wire is wound upon the inside of a highly refractory wall embedded in and entirely covered with a very high fusing porcelain. In order that the wire will not break out of the porcelain investment by repeated expansion and contractions, it must be wound in very short curves somewhat "S"-shaped, and not more than half an inch in length. If this can be done, then a "foll proof" electric oven can be put upon the market. Its electrical resistance being about half that of platinum, it should therefore be half the cross section when substituted for platinum.

As before stated, tungsten is not acted upon by ordinary reagents. It is only soluble in hot nitro-hydrofluoric acid. This being the case, it can be substituted in many places for platinum.

The third property of this metal is its hardness. We are surprised that a metal of such high fusing point should possess this property. In the wire form it is about like annealed piano wire. This property and its resistance to the action of ordinary acids makes tungsten wire a valuable instrument for using acids in the pulp canal. A tungsten broach does not become brittle and may be repeatedly bent without breaking.

The hardness and high fusing point of this metal makes it of high value for the contact point of induction coils and for X-Ray tube targets. The density of wrought tungsten is 19.3, while that of lead is 11.5. It is destined to play an important role in the field of contact making devices. This is due to its high melting point which prevents them from welding together; to its heat conductively (about twice that of platinum; which tends to keep down local temperature rise; and to its hardness, which enables it to stand repeated impact without flattening out. "The natural assumption would be that this metal under the heat of even minute arcs would oxidize at the point where arcing takes place, and that non-conducting layers would thus be formed, which would produce a high and variable resistance. But several things intervene to prevent this. First, there is the relatively good heat conductively of the dense form of this metal which distributes the heat. Then the much less cost of this metal as compared with platinum, makes possible the use of larger contact masses, which again militates against strong local heating. Finally, there is the fact that the oxides, which form in the presence of a limited amount of oxygen are conductive."

An exceptionally interesting application of tungsten is for the target of an X-Ray tube. Heretofore, platinum has been used for this purpose, but while this was the best, it possessed two properties which were decidedly objectionable—its softness and its property of occluding hydrogen. In an X-Ray tube, the electrodes are shot out from the concave cathode at the target, which standing at an angle of 45 degrees as related to the cathode, reflects the X-Ray out of the tube. Now the closer the focus of the rays upon the target, the better the rays. The impaction of the rays upon the target quickly heats the same, and it is here that the hardness and conductivity of tungsten is of very great value over platinum, for it permits of a finer focus and with less heat.

In the latest X-Ray apparatus, as much as six horse-power of energy are used, and where this is all centered upon a very small point of the target (the smaller the better) it is evident that much heat is generated. Where platinum is used, although it has a high melting point, the highest convergence of the rays was not allowable. Here the high value of tungsten is again noticeable.

The maintenance of a uniform degree of vacuum is of the utmost importance in X-Ray work, and the property of platinum for occluding gases and freeing them when heated has always been a troublseome feature with large platinum targets. Here again, we have another advantage of tungsten over platinum, for tungsten occludes gases least of all metals.

If the coefficient of expansion is the same as that of porcelain, as I think it will be found to be, an oxidation can be prevented by the us of a hydrogen vapor (if hydrogen should not effect the color of porcelain), then we can have porcelain teeth and crowns with pins of steel-like hardness.

At the present time, this metal is controlled by the General Electric Co., and as yet no market price has been placed upon it. But when it can be had, I am informed that it will not be an expensive metal as compared with platinum. I am indebted to W. D. Coolidge of the General Electric Co. for much of the data in this article.—*Dental Brief.*

